# Effects of a Meal Replacement on Body Composition and Metabolic Parameters among Subjects with Overweight or Obesity

**DOI:** 10.1155/2018/2837367

**Published:** 2018-12-26

**Authors:** Xiaohui Guo, Yifan Xu, Hairong He, Hao Cai, Jianfen Zhang, Yibin Li, Xinyu Yan, Man Zhang, Na Zhang, Rolando L. Maddela, Jessie Nicodemus-Johnson, Guansheng Ma

**Affiliations:** ^1^Department of Nutrition and Food Hygiene, School of Public Health, Peking University, 38 Xue Yuan Road, Haidian District, Beijing 100191, China; ^2^USANA Health Sciences Inc., 3838 W Parkway Boulevard, West Valley City, UT 84120, USA; ^3^Beijing Key Laboratory of Toxicological Research and Risk Assessment for Food Safety, School of Public Health, Peking University, 38 Xue Yuan Road, Haidian District, Beijing 100191, China

## Abstract

Meal replacement plans are effective tools for weight loss and improvement of various clinical characteristics but not sustainable due to the severe energy restriction. The aim of the study was to evaluate the impact of meal replacement, specifically 388 kcal in total energy, on body composition and metabolic parameters in individuals with overweight and obesity from a Chinese population. A parallel, randomized controlled trial was performed with 174 participants (ChiCTR-OOC-17012000). The intervention group (*N*=86) was provided with a dinner meal replacement, and the control group (*N*=88) continued their routine diet as before. Body composition and blood parameters were assessed at 0, 4, 8, and 12 weeks. A post hoc analysis (least significant difference (LSD) test), repeated measurements, and paired *T*-test were used to compare each variable within and between groups. Significant (*p* < 0.001) improvements in body composition components were observed among the intervention group, including body weight (−4.3 ± 3.3%), body mass index (−4.3 ± 3.3%), waist circumference (−4.3 ± 4.4%), fat-free mass (−1.8 ± 2.9%), and body fat mass (−5.3 ± 8.8%). Body composition improvements corresponded with significant metabolic improvements of blood glucose (−4.7 ± 9.8%). Further improvements in visceral fat area (−7.7 ± 10.1%), accompanying with improvements in systolic (−3.7 ± 6.9%) and diastolic (−5.3 ± 7.7%) blood pressure, were only found in male subjects. To conclude, meal replacement intake with 388 kcal in total energy at dinner time for 12 weeks contributed to improvement in body composition and clinically significant metabolic parameters in both male and female participants with overweight/obesity. Additionally, glucose and blood pressure reduction were gender-specific highlighting the importance of gender stratification for design of nutritional intervention studies for improvement of health.

## 1. Introduction

Obesity has increased dramatically worldwide over the past few decades [1]. According to the World Health Organization, more than one-third of adults in China are overweight, while 7% of adults are obese [2], with some provinces reaching epidemic proportions [3]. Chronic obesity increases individual risks for many diseases such as cardiovascular disease, type 2 diabetes, and hypertension [4, 5], which are also increasing in prevalence among Chinese populations at an alarming rate [6]. From a health-care perspective, addressing overweight and obesity is an important strategy that contributes to the prevention or reduced risk of developing these diseases within the Chinese population [7].

A chronic imbalance between energy intake and energy expenditure plays an important role in the development of obesity. Long-term negative energy balance is necessary for individuals with overweight and obesity to lose weight. Energy-restricted meal replacements are a safe and effective strategy for weight control that has been implemented in many studies [8, 9]. In addition, lifestyle changes, accompanied by partial or whole meal replacements, in which some aspects of the individuals' daily routine are altered may be more preferable. However, physiological response to specific meal replacement diets is heterogeneous and may vary by individual or population. Specifically, there are few studies looking at the effects of low-calorie meal replacement in Chinese populations, for which environmental, nutritional, and physiological differences are known to exist [10].

Weight-control and weight-loss studies performed with meal replacements were always accompanied by very low calorie intake (<800 kcal/day) or low calorie intake (800–1500 kcal/day), which improved weight reduction rapidly but not sustainably [11, 12]. A key problem is the high attrition rate. A meta-analysis and pooling analysis reported a 16% and 47% drop out rate after 3-month and 1-year intervention with low-calorie meal replacement, respectively [9]. Another meta-analysis shows (49%) studies reported 30% attrition in commercial weight-loss programs [13]. One of the possible explanations is that very low calorie/low calorie intake is not easy to maintain in a real-world setting. Mild energy-restricted meal replacement may be able to solve this problem, but few studies have reported significant results with this type of diet plan.

Inverse associations between weight loss, normally expressed by reduction in BMI, and metabolic parameters in individuals with overweight and obesity have been found in previous studies [14, 15]. Abdominal fat reduction has been shown to be a better predictor of metabolic risk than BMI. Generally, abdominal fat was determined by waist circumference and waist-to-hip height ratio; however, waist circumference is not precise enough because it is a function of both the subcutaneous adipose tissue and visceral adipose tissue compartments [16, 17]. Actually, visceral fat area, another indicator of abdominal fat, shows better associations with metabolic parameters. This may be explained by a higher amount of visceral fat related to extraadipocyte fat storage, reduced insulin sensitivity and increased proinflammatory adipokine concentrations [18]. However, little is known about the effect of meal replacement on visceral fat in a clinical trial setting, and there is a need to explore the effects of weight control by meal replacement on visceral fat and body composition [18, 19].

Despite the fact that gender-specific physiological differences may result in variable responses to weight loss, they are rarely highlighted in meal replacement weight-loss studies. There are a number of characteristics that differ between men and women during weight-loss programs due to the difference in fat distribution and the hormone level [18]. Women tend to have more fat on subcutaneous area, and men are more likely to exhibit central obesity [20]. Men tend to lose weight faster, although both genders lose weight overall [21]. Also, men and women exhibit different attitudes and behaviors surrounding weight and weight management, which may contribute to variation in weight-loss program efficacy [22]. The gender differences in diet and body composition mentioned above, as well as many others, are likely to play a role in meal replacement processing and manifestation of clinically relevant health improvements, such as reductions in blood pressure, blood lipids, etc. Although many studies have addressed gender in weight loss, gender assessment in meal replacement trials is limited [22, 23].

The aim of the current study was to evaluate the effects of a mild restricted meal replacement on body composition and metabolic parameters in male and female subjects with overweight or obesity from a Chinese population. We hypothesized that a slight reduction in energy intake by partial meal replacement can achieve clinically relevant weight loss and metabolic profile improvements over a 12-week intervention period and that a subset of these effects will vary by gender.

## 2. Materials and Methods

### 2.1. Ethics Statement

The Ethics Committee of Peking University Health Science Center approved the study protocol on 6 July 2017. The authors confirm that all ongoing and related trials for this drug/intervention are carried out by following the rules of the Declaration of Helsinki of 1964 and registered (ChiCTR-OOC-17012000).

### 2.2. Subjects

We sent recruitment advertisement to free-living communities in and around the Beijing area, and all potential participants were asked to read and sign an informed consent as well as complete a basic screening questionnaire which aimed at assessing eligibility. Specifically, inclusion criteria were (1) generally healthy participants with BMI >24 kg/m^2^, aged between 18 and 55 years; (2) currently, not participated in any weight-loss activity; (3) did not have any allergies to any of the known food ingredients; (4) currently were not pregnant or did not intend to be pregnant. Exclusion criteria were (1) diagnosis of cognitive impairment, schizophrenia, or depression by a physician; (2) alcoholism, defined as 61 g alcohol drinks per day for male and 41 g alcohol drinks per day for female; (3) equipped with pacemaker or other internal electronic medical device; (4) previously underwent weight-loss surgery; and (5) irregular diet and work such as night shift.

The sample size was calculated based on an intervention and control group difference in weight change of 2.4 kg, approximately 3% for individual with 80 kg, and a 7.5 kg SD of weight change [24]. The estimated 154 subjects provided a power of 80% to detect this difference in weight change at a two-tailed significance level of 0.05. Assuming that the attrition rate is 20% after the intervention, the final sample size was estimated to 185 individuals.

### 2.3. Study Design

The study was a paralleled, randomized controlled clinical trial. After screening and selection, participants were randomized into 2 groups, the meal replacement group (intervention group) and the routine diet group (control group). The intervention group was advised to consume one liquid meal replacement (Nutrimeal©, Fibergy©, USANA Health Sciences Inc.), which contains 22.6 g protein, 11.1 g fat, 39.3 g carbohydrate, 20.9 g dietary fiber, and 388 kcal in total energy at dinner time during the intervention, and the control group was advised to follow a routine Chinese dinner as before. Additional nutrient profile for meal replacement can be found in Supplementary Table S1. The summarized nutritional profiles between the control and intervention can be found in Table S2. Individuals were advised to continue their regular physical activity regimen. Participants were invited back to reservation location for assessment of body composition and blood parameters at 0 (initial), 4, 8, and 12 (postintervention) weeks during the intervention. After each visit, the intervention group was given sufficient meal replacement sachets to last until the next visit at no charge. If a participant failed to attend a scheduled appointment, they were contacted, and if participants were unable or unwilling to regularly attend the scheduled appointments after study initiation, they were considered drop-outs.

### 2.4. Dietary Assessment

Dietary habits were assessed through a self-administered 77-item Food Frequency Questionnaire (FFQ) at the first and last visit. Information about lifestyle, health condition, education, history of illnesses, and medication use was collected by a brief self-administered 17-item general questionnaire. Physical activity was evaluated by a self-administered 24-item questionnaire.

### 2.5. Body Composition

Body composition was measured at baseline and at 4-week intervals throughout the study by using multifrequency bioelectrical impedance analysis with 8-point tactile electrodes (InBody 720; Biospace, Seoul, Korea) [25]. Bioelectric impedance was measured within 1-2 minutes with the subject standing in her/his bare feet and grasping the hand electrodes with arms in the vertical position. Height was measured using a standard wall-mounted stadiometer to the nearest 0.5 cm. BMI was calculated as weight in kilograms divided by height in square meters. Overweight is defined as a condition where a subject has a BMI between 24 and 28 kg/m^2^, and obesity is defined as a condition where a subject has a BMI higher than 28 kg/m^2^, according to current definitions in China [26]. Body composition parameters also include waist-to-hip ratio (WHtR), intracellular water (ICW), extracellular water (ECW), total body water (TW), protein, minerals, fat-free mass (FFM), body fat mass (BFM), visceral fat area (VFA), body cell mass (BCM), and basal metabolic rate (BMR).

### 2.6. Metabolic Parameters Measurements

Metabolic parameters measured at initial and postintervention visit included systolic blood pressure (SBP), diastolic blood pressure (DBP), fasting glucose (GLU), total cholesterol (TC), triglycerides (TG), high-density lipoprotein cholesterol (HDL-C), and low-density lipoprotein cholesterol (LDL-C). For the measurement of blood pressure, a validated semiautomatic sphygmomanometer (Omron HEM-705CP) was used by trained nurses. Two measurements were taken at 5-minute intervals with participants in a seated position. Data were reported as an average of 2 measurements [27]. Plasma glucose, total cholesterol, and triglyceride concentrations were measured using standard enzymatic automated methods. Levels of HDL cholesterol were measured by an enzymatic procedure after precipitation, and LDL cholesterol was estimated by the Friedewald formula [28].

In the current study, hypertension is defined as systolic blood pressure (SBP) higher than 140 mmHg or diastolic blood pressure (DBP) higher than 90 mmHg [29]. Diabetes is defined as self-reported history of diabetes or fasting plasma glucose (FPG) ≥7.8 mmol/L or 2 h plasma glucose ≥11.1 mmol/L on 75 g oral glucose tolerance test [30]. Dyslipidemia is defined by self-reported history of dyslipidemia or elevations in levels of TC, LDL-C, TG higher than 6.22 mmol/L, 4.14 mmol/L, 2.26 mmol/L, respectively, or HDL-C less than 1.04 mmol/L [31].

### 2.7. Statistical Analysis

To ensure data met assumptions of parametric tests, data were assessed for outliers, homoscedasticity, and normality using Kolmogorov and Levene tests. Nutrients intake and food consumption according to the FFQs were assessed at baseline and postintervention. A post hoc analysis (least significant difference (LSD) test), repeated measurements, and paired *T*-test were used to compare each variable within and between groups.

All analyses were performed using SPSS software V24.0 (SPSS Inc., Chicago, IL, USA). Results were expressed as mean ± SD for continuous variables or percentages for categorical variables. All statistical tests were two-tailed, and the threshold for significance level was *p* < 0.05.

## 3. Results

### 3.1. Flowchart

**Figure 1:**
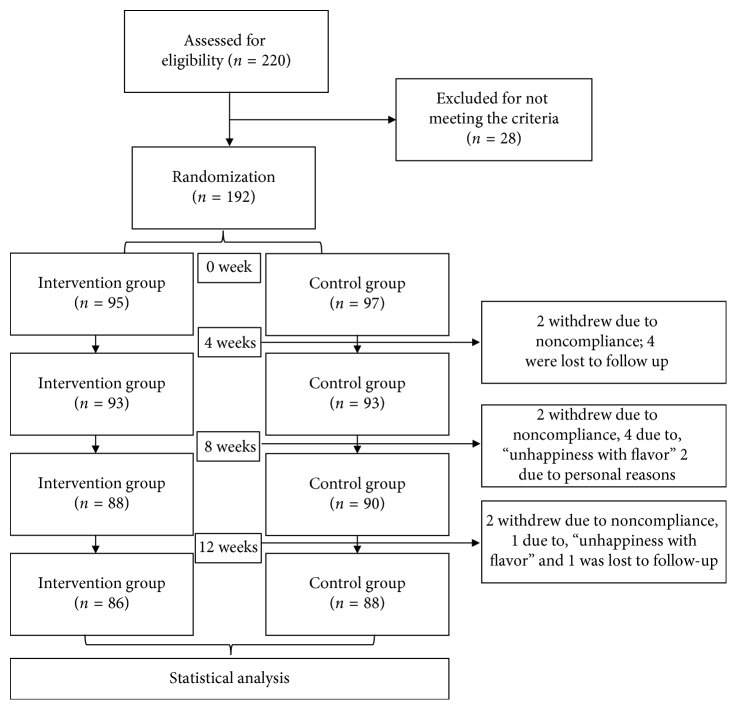
Flowchart of study participants.

Figure 1 represents a flowchart depicting study design and participant dropout rate. A total of 220 subjects were recruited from Beijing and surrounding areas. Twenty-eight were excluded because they did not meet the inclusion criteria. After the intervention, 18 withdrew for various reasons: 5 because they were not able to meet the scheduled appointment, 8 due to noncompliance, 3 because they were unhappy with the flavor, and 2 due to personal reasons; hence, a total of 174 participants were finally included.

### 3.2. Baseline Characteristics

The baseline characteristics of participants are shown in Table 1. Eighty-six individuals were assigned to the intervention groups (42 male and 44 female) and eighty-eight to the control groups (38 male and 50 female). Intervention and control groups were not found to be significantly different for any of the parameters measured. Male subjects had a mean age of 38.9 ± 6.5 and 38.0 ± 6.6 years, and body weight of 89.9 ± 12.5 and 89.2 ± 10.2 kg for intervention and control groups, respectively. Of the participants, 61.9% and 43.2% had hypertension, 10% and 2.7% had diabetes, 26.2% and 39.5% had dyslipidemia, and 11.9% and 10.5% were current smokers in the intervention and control groups, respectively. Female participants had a mean age of 39.4 ± 7.9, 37.0 ± 7.8 years and body weight of 73.3 ± 7.9, 73.4 ± 8.5 kg for the intervention and control group, respectively. Moreover, 22.7% and 18.0% had hypertension, 2.4% and 4.0% had diabetes, 13.6% and 24.0% had dyslipidemia, and 9.1% and 4.0% were current smokers in the intervention and control groups, respectively.

### 3.3. Improvement in Body Composition Parameters

Relevant body composition parameters (BW, BMI, WC, WHtR, FFM, BFM, VFA, and BCM) stratified by time point and treatment group are shown in Table 2. Additional body composition parameters (ICW, ECW, TW, protein, minerals, and BMR) measured are shown in Table S3. In the intervention group, significant (*p* < 0.001) reductions were found in BW, BMI, WC, ICW, ECW, TW, protein, minerals, FFM, BFM, BCM, and BMR in the combined analysis. Similar results were observed after gender stratification. BFM was significantly decreased in both genders; however, the percent decrease was double in males (−7.7%) relative to females (−3.2%). Additionally, VFA was only significant in males (*p* < 0.001) relative to females (*p*=0.012). This male-specific reduction in VFA was 4.3 times greater than the modest reduction observed in females. The absolute FFM decreased significantly during the intervention in both genders, while the relative FFM increased from 69.5% ± 3.5% to 71.4% ± 4.7% (*p* < 0.001 in male and from 60.5% ± 4.7% to 62.1% ± 5.4%, *p* < 0.001) in females, respectively. Consistently, absolute reduction in BCM was observed after intervention for both genders, while a slight increase in relative BCM was shown in the intervention group (from 39.2% ± 3.0% to 40.4% ± 3.6% in female, *p* < 0.001; from 45.5% ± 2.6% to 47.0% ± 3.6% in male, *p* < 0.001). Among the intervention group, the ∆FFM: ∆BW was 0.33 ± 1.83 overall and 0.47 ± 2.43 and 0.22 ± 1.14 for males and females, respectively.

In the control group, significant, although modest (<1%), changes were found in BW, BMI, WC, FFM, BCM, ICW, protein, minerals, and BMR in the combined analysis. ICW, TW, and BMR were not significant in the combined analysis. WHtR, BFM, and VFA increased significantly for both genders when compared at baseline and postintervention. Furthermore, BMI, BFM, and VFA in male and WC in both genders showed significant differences between the intervention and control group at the end of intervention.

### 3.4. Improvement in Metabolic Parameters

Metabolic parameters stratified by time point and the treatment group are shown in Table 3. Systolic blood pressure improvements were observed among males (mean reduction 3.7%) but not females (*p*=0.678; mean reduction 1.3%). Also, diastolic blood pressure improvements were observed among males (mean reduction 5.3%) but not females (*p*=0.060; mean reduction 2.5%). Among the intervention group, significant reductions were observed for glucose in the combined dataset. The mean improvement in glucose levels was 4.7%. Similar trends in improvement were observed separately in male and female glucose data. For the control group, increment in SBP (mean increment 3.2%), HDL (mean increment 6.4%), and LDL-C (mean increment 6.7%) was found in females, and reduction in DBP (mean reduction 3.9%) and TG (mean reduction 11.1%) and increment in LDL-C (mean increment 4.5%) were found in males. No significant difference was found between the intervention group and the control group at the 12thweek.

### 3.5. Nutrients and Food Intake

Table S2 shows nutrients intake and food consumption during the intervention. At baseline, total daily energy intakes were 2162 ± 390 kcal, 2108 ± 306 kcal for males, 1832 ± 294 kcal, 1911 ± 399 kcal for females in the intervention groups and control group, respectively. There is no difference between the intervention group and the control group at baseline (*p*=0.495 for male; *p*=0.284 for female). However, energy intakes decreased by 220 kcal/d in the intervention group but not in the control group after intervention. Additionally, physical activity, expressed as MET-min per day, did not show any difference within and between groups (*p*=0.639).

There is a significant increase in total protein and fiber intake in the intervention group relative to controls. This may partially be explained by consumption of the meal replacement which contains large amounts of fiber and protein (*p* < 0.001). Additionally, we observed a significant reduction in carbohydrate intake in the intervention group relative to the control.

As for food intakes, differences were found in milk, meat, legume, cereals, and beverage intake after intervention between the two groups. Significant reductions (*p* < 0.001) were shown in milk, meat, legume, and beverage intakes in the intervention group.

## 4. Discussion

A 12-week meal replacement with mild caloric restriction study conducted in a group of Chinese participants with overweight and obesity showed significant reduction or improvement in 14 body composition parameters as well as 3 out of 7 metabolic parameters assessed. Our findings implemented a slight energy reduction design, likely making it more acceptable and sustainable to participants in the context of lifestyle modifications [13].

Associations between weight loss and improvements in metabolic parameters have been demonstrated by numerous studies [9, 14, 32]. Although weight loss reported in the present study did not reach clinical significance, defined as 5% reduction in weight over a period of at least 12 weeks, even small increments of weight loss have been shown to be important for the prevention of cardiovascular diseases [13, 33] and thus confer meaningful health benefits, such as improvements in glucose and blood pressure [34]. Furthermore, it has been suggested that the threshold for clinically significant weight loss is variable depending on the clinical phenotypes in question. For example, while a 5% weight loss has been suggested to be a marker of clinically significant changes in blood pressure, HDL, and LDL, a 3% weight loss has been shown to be clinically relevant for blood sugar and triglyceride levels [35]. Regardless of the weight loss cut-off, in the present study, the overall loss of 4.3% body weight coincides with clinically significant improvements in blood pressure (males only) and glucose (males and females) levels. Moreover, due to the moderate duration of this study (only 12 weeks), we believe additional studies with longer durations will reach the 5% benchmark, resulting in greater improvements in clinical risk factors.

Maximizing fat loss while preserving muscle mass is the central goal of obesity treatments. As such, attention to body composition parameters such as free fat mass are critical to the assessment of the clinical utility of weight-loss programs, wherein the ratio of free-fat mass loss to weight loss may serve as a biomarker of clinical efficacy [36, 37]. In the current study, the modest weight loss observed in this program is associated with ∆FFM/∆BW ratio of 0.3, consistent with the commonly reported ratio of 0.25 lending to the efficacy of this weight-loss approach. In addition, while the absolute FFM and BCM decreased in the end of intervention, the relative FFM and BCM increased, indicating a satisfactory overall development of muscle mass despite weight reduction. Our finding is in line with a long-term weight-loss maintenance by a meal replacement program [38] and concordant with results of a similar study in a 40-week randomized, controlled clinical trial, which aimed at evaluating the impact of a portion-controlled meal replacement diet plan on body weight and body composition [39].

Gender is known to modify both nutritional intake and phenotypic response [40–42]. Therefore, studying the difference between men and women in response to interventions is essential for providing optimal care. The present study demonstrated modest improvements in VFA (4.5% reduction); however, when stratified by gender, the majority of this effect was observed in the male subset of our population (7.7% reduction), while females demonstrated a marginal reduction (1.8%). Gender differences in VFA may be due to sex hormone-driven differences in body composition and fat distribution with men having a more central distribution of fat, i.e., preferentially storing and losing fat in visceral deposits, while women of child-bearing age have more subcutaneous adipose tissue deposits [43].

Male-specific effects were extended to SBP measurements in which males demonstrated a 3.7% improvement relative to 1.3% reduction in females. This result is consistent with previous studies that demonstrate VFA is a better predictor of metabolic risk parameters, such as SBP [16, 17]. The observed reductions in body weight and visceral fat are known to contribute to correlative reductions in blood pressure, as observed in this study, might through improvement in glucose tolerance [44]. The gender-specific effects in VFA and blood pressure in response to weight loss suggest men and women should adopt different weight-loss strategies.

Notably, although VFA improvement and blood pressure reduction were found in males only, improvements in overall weight parameters (BMI, BW, and WC; 4.3%) coincided with a 4.7% reduction in blood glucose levels, irrespective of gender thus identifying two different body composition parameters that are accompanied with different cardio metabolic risk parameters in the present study. Therefore, our findings highlight the fact that different body composition parameters may be better predictors of specific clinical phenotypes. The gender-neutral results correspond with those of other published studies. For example, a cross-sectional study also found inverse associations between visceral fat and blood glucose, and a longitudinal study showed changes in VFA were significantly correlated with changes in fasting plasma glucose [45, 46].

Some limitations of this study should be noted. First, the length of the intervention was only 12 weeks, a longer duration may be more informative and enhance nominally significant associations in light of our modest caloric intervention. Our short-term intervention cannot assess the long-term intervention associations with body composition and metabolic parameters. Second, the sample size is relatively small under stratified analysis; however, our results are robust to multiple testing, and the paired longitudinal design of the study increases our power to detect reported associations. Third, food and nutrient intake were evaluated by self-administered food frequency questionnaire using food recall methods, which maybe not be precise enough for nutritional inference. Fourth, the observed physiological improvements may result from alteration to meal patterns or specific micronutrient intake; however, this is out of the scope of the current study.

## 5. Conclusions

Overall, our study reports the results of a 12-week randomized controlled intervention in a Chinese population with overweight and obesity. We demonstrate clinically relevant improvements in metabolic parameters with modest weight loss (<5%). Additionally, we demonstrate both gender-specific and gender-independent body composition improvements that accompanied independent metabolic parameters. For future research, gender-specific associations between weight loss in subcutaneous fat and visceral fat and improvement in metabolic parameters need to be further explored. Finally, compared with other energy restricted programs, our trial was conducted based on a slight energy reduction design, making it more acceptable and sustainable in the context of lifestyle modifications.

## Figures and Tables

**Table 1:** Characteristics of participants at baseline.

	Male		*p*	Female		*p*
	Intervention group	Control group		Intervention group	Control group	
No. of subjects	42	38		44	50	0.454
Age (y), mean (SD)	38.9 ± 6.5	38.0 ± 6.6	0.539	39.4 ± 7.9	37.0 ± 7.8	0.134
Body weight (kg), mean (SD)	89.9 ± 12.5	89.2 ± 10.2	0.916	73.3 ± 7.9	73.4 ± 8.5	0.852
BMI (kg/m^2^), mean (SD)	29.8 ± 3.1	28.7 ± 3.0	0.474	28.9 ± 3.2	28.7 ± 3.0	0.909
Systolic BP (mmHg), mean (SD)	136.6 ± 16.4	132.6 ± 12.3	0.104	121.7 ± 15.2	120.1 ± 17.1	0.914
Diastolic BP (mmHg), mean (SD)	89.6 ± 11.6	88.6 ± 11.9	0.445	79.5 ± 11.0	77.9 ± 12.9	0.717
Hypertension, *n* (%)	26 (61.9)	16 (43.2)	0.097	10 (22.7)	9 (18.0)	0.463
Diabetes, *n* (%)	4 (10)	1 (2.7)	0.204	1 (2.4)	2 (4.0)	0.651
Dyslipidemia, *n* (%)	11 (26.2)	15 (39.5)	0.152	6 (13.6)	12 (24)	0.221
Smoking status, *n* (%)			0.629			0.280
Current	5 (11.9)	4 (10.5)		4 (9.1)	2 (4.0)	
Former	3 (7.1)	1 (2.6)		0 (0)	0 (0)	
Never	34 (81)	33 (86.8)		40 (90.9)	48 (96.0)	
Medication, *n* (%)						
Aspirin	0 (0)	0 (0)		0 (0)	0 (0)	
Antihypertension	7 (16.7)	3 (7.9)	0.200	1 (2.3)	2 (4.0)	0.548
Hypolipidemic drugs (%)	3 (7.1)	2 (5.3)	0.548	0 (0)	2 (4.0)	0.280
Insulin	0 (0)	0 (0)		0 (0)	0 (0)	
Oral hypoglycemic drugs	1 (2.4)	1 (2.6)	0.728	1 (2.3)	1 (2.3)	0.720
Vitamins	4 (9.5)	4 (10.5)	0.586	5 (11.4)	4 (8.0)	0.418
Minerals	3 (7.1)	3 (7.9)	0.613	3 (6.8)	4 (8.0)	0.572
Education level (%)			0.593			0.496
University	33 (78.6)	30 (78.9)		36 (81.8)	42 (84)	
High school	9 (21.4)	8 (21.1)		8 (18.2)	8 (16.0)	
Primary school	0 (0)	0 (0)		0 (0)	0 (0)	
Marital status (%)			0.613			0.144
Single	3 (7.1)	3 (7.9)		3 (6.8)	8 (16.0)	
Married	39 (92.9)	35 (92.1)		41 (93.2)	42 (84)	
Widowed	0 (0)	0 (0)		0 (0)	0 (0)	

BMI: body mass index; BP: blood pressure. Data are given as mean (SD) for continuous variables and percentages for categorical variables; *p* < 0.05 indicates statistical significance. *p* values calculated by analysis of variance or *χ* ^2^ tests.

**Table 2:** Characteristics of body composition before and after intervention^a^.

	Intervention group (*n*=86)				Mean percent reduction (%)	*p* for trend^b^	*p* ^c^	Control group (*n*=88)				Mean percent reduction (%)	*p* for trend^b^	*p* ^c^	*p* ^d^
	Time 1	Time 2	Time 3	Time 4				Time 1	Time 2	Time 3	Time 4				
BW (kg)															
Male	89.9 ± 12.5	88.3 ± 11.8	87.6 ± 12.2	85.9 ± 12.4	−4.5 ± 3.4	<0.001	<0.001	89.2 ± 10.2	90.1 ± 10.0	90.0 ± 9.8	89.6 ± 9.9	0.2 ± 3.1	0.013	0.353	0.149
Female	73.3 ± 7.9	72.6 ± 8.3	71.6 ± 7.3	70.1 ± 8.4	−4.2 ± 3.2	<0.001	<0.001	73.4 ± 8.5	74.1 ± 9.0	73.9 ± 9.5	72.9 ± 9.0	−0.6 ± 2.9	<0.001	0.145	0.101
Total	81.0 ± 13.2	79.8 ± 12.8	78.9 ± 12.7	77.5 ± 13.0	−4.3 ± 3.3	<0.001	<0.001	80.1 ± 12.1	80.9 ± 12.3	80.7 ± 12.4	80.0 ± 12.5	−0.3 ± 3.0	<0.001	0.698	0.137

BMI (kg/m^2^)															
Male	29.8 ± 3.1	29.3 ± 2.9	29.1 ± 3.1	28.5 ± 3.2	−4.5 ± 3.4	<0.001	<0.001	30.1 ± 2.6	30.4 ± 2.5	30.4 ± 2.4	30.3 ± 2.5	0.2 ± 3.1	0.012	0.117	0.013
Female	28.9 ± 3.2	28.6 ± 3.3	28.2 ± 3.2	27.7 ± 3.3	−4.2 ± 3.2	<0.001	<0.001	28.7 ± 3.0	29.0 ± 3.1	28.9 ± 3.3	28.5 ± 3.2	−0.6 ± 2.9	<0.001	0.134	0.150
Total	29.3 ± 3.2	28.9 ± 3.1	28.6 ± 3.2	28.1 ± 3.3	−4.3 ± 3.3	<0.001	<0.001	29.3 ± 2.9	29.6 ± 3.0	29.5 ± 3.0	29.3 ± 3.0	−0.3 ± 3.0	<0.001	0.576	0.009

WC (cm)															
Male	102.3 ± 7.4	100.2 ± 7.0	99.1 ± 6.6	97.4 ± 6.8	−4.7 ± 3.7	<0.001	<0.001	102.8 ± 7.2	103.1 ± 7.0	103.0 ± 7.0	103.5 ± 7.2	0.7 ± 2.8	0.392	0.487	<0.001
Female	93.1 ± 7.5	92.1 ± 8.4	90.7 ± 8.6	89.5 ± 8.9	−3.9 ± 5.0	<0.001	<0.001	93.1 ± 7.7	93.3 ± 6.9	92.9 ± 7.7	93.6 ± 7.0	0.7 ± 3.1	0.409	0.186	0.014
Total	97.5 ± 8.8	96.0 ± 8.7	94.7 ± 8.7	93.0 ± 8.9	−4.3 ± 4.4	<0.001	<0.001	97.3 ± 8.9	97.5 ± 8.4	97.2 ± 8.9	97.9 ± 8.6	0.7 ± 2.9	0.134	0.042	0.001

WHtR															
Male	0.97 ± 0.06	0.97 ± 0.06	0.97 ± 0.07	0.97 ± 0.06	−0.3 ± 3.0	0.361	0.600	0.96 ± 0.06	0.98 ± 0.06	0.98 ± 0.06	0.99 ± 0.09	3.6 ± 6.1	0.006	0.010	0.203
Female	0.93 ± 0.05	0.94 ± 0.05	0.94 ± 0.05	0.95 ± 0.06	2.5 ± 3.1^*∗*^	<0.001	<0.001	0.92 ± 0.05	0.95 ± 0.05	0.95 ± 0.06	0.97 ± 0.05	4.1 ± 2.7	<0.001	<0.001	0.618
Total	0.95 ± 0.06	0.96 ± 0.06	0.95 ± 0.06	0.96 ± 0.06	1.3 ± 3.3	0.002	0.002	0.94 ± 0.06	0.96 ± 0.06	0.96 ± 0.06	0.98 ± 0.07	3.9 ± 4.5	<0.001	<0.001	0.507

FFM (kg)															
Male	62.2 ± 6.8	61.1 ± 6.4	60.7 ± 6.6	60.9 ± 6.3	−2.0 ± 2.2	<0.001	<0.001	60.5 ± 6.3	60.6 ± 6.8	60.0 ± 6.9	60.7 ± 7.2	0.3 ± 4.0	0.225	0.560	0.987
Female	44.2 ± 4.3	43.2 ± 4.3	43.1 ± 4.1	43.4 ± 4.4	−1.7 ± 3.4	<0.001	<0.001	44.2 ± 4.2	43.9 ± 4.6	43.8 ± 4.6	43.8 ± 4.6	−0.8 ± 2.9	0.050	0.078	0.551
Total	52.5 ± 10.6	51.5 ± 10.4	51.2 ± 10.3	51.5 ± 10.3	−1.8 ± 2.9	<0.001	<0.001	51.0 ± 9.6	50.9 ± 10.0	50.6 ± 9.8	51.0 ± 10.2	−0.3 ± 3.4	0.085	0.650	0.940

BFM (kg)															
Male	27.7 ± 6.8	27.2 ± 6.5	26.0 ± 7.4	25.7 ± 7.1	−7.7 ± 9.1	<0.001	<0.001	28.7 ± 6.8	29.5 ± 6.5	28.7 ± 6.9	29.9 ± 7.3	3.2 ± 13.2	0.021	0.025	0.022
Female	29.1 ± 5.9	29.3 ± 6.1	27.5 ± 5.9	28.2 ± 6.3	−3.2 ± 7.1^*∗*^	<0.001	0.005	29.2 ± 5.8	30.2 ± 6.1	29.1 ± 6.2	30.1 ± 6.1	3.2 ± 6.8	0.001	0.002	0.112
Total	28.5 ± 6.3	28.3 ± 6.3	26.8 ± 6.6	27.1 ± 6.8	−5.3 ± 8.3	<0.001	<0.001	29.0 ± 6.2	29.9 ± 6.2	29.0 ± 6.5	30.0 ± 6.6	3.2 ± 10.0	<0.001	<0.001	0.005

VFA (cm^2^)															
Male	124.7 ± 33.4	122.3 ± 32.7	117.5 ± 37.5	115.7 ± 36.0	−7.7 ± 10.1	<0.001	<0.001	128.2 ± 36.0	133.6 ± 36.0	131.1 ± 38.6	137.5 ± 44.9	5.7 ± 16.9	0.021	0.012	0.036
Female	144.9 ± 32.5	148.6 ± 34.1	138.7 ± 35.1	143.2 ± 37.5	−1.8 ± 10.4^*∗*^	0.012	0.389	145.2 ± 30.7	152.9 ± 32.8	147.5 ± 34.3	154.2 ± 33.0	6.5 ± 8.5	<0.001	<0.001	0.104
Total	135.6 ± 34.2	136.5 ± 35.7	128.9 ± 37.5	130.5 ± 39.1	−4.5 ± 10.6	<0.001	<0.001	138.0 ± 33.9	144.8 ± 35.3	140.6 ± 36.8	147.2 ± 39.1	6.2 ± 12.7	<0.001	<0.001	0.008

BCM (kg)															
Male	40.7 ± 4.5	40.1 ± 4.2	39.8 ± 4.3	40.1 ± 4.2	−1.4 ± 2.1	<0.001	<0.001	39.6 ± 4.1	39.8 ± 4.4	39.4 ± 4.5	40.0 ± 4.7	0.7 ± 4.2	0.132	0.257	0.960
Female	29.6 ± 2.8	28.1 ± 2.8	28.0 ± 2.6	28.2 ± 2.8	−1.2 ± 3.5	<0.001	0.014	28.7 ± 2.8	28.6 ± 3.0	28.5 ± 3.0	28.6 ± 3.0	−0.3 ± 2.8	0.271	0.473	0.469
Total	34.2 ± 7.1	33.6 ± 6.9	33.5 ± 6.9	33.7 ± 6.9	−1.3 ± 2.9	<0.001	<0.001	33.3 ± 6.4	33.3 ± 6.7	33.1 ± 6.5	33.4 ± 6.8	0.1 ± 3.5	0.077	0.524	0.980

^a^Data are given as means (SD); ^b^data were analyzed by repeated measurements; ^c^data analyzed by paired-samples *T*-test between time 1 and time 4; ^d^data analyzed by *T*-test between the intervention group and control group at time 4. Asterisk shows difference between genders. BW: body weight; BMI: body mass index; WC: waist circumference; WHtR: waist-to-hip ratio; FFM: fat-free mass; BFM: body fat mass; VFA: visceral fat area; BCM: body cell mass. *p* < 0.05 indicates statistical significance.

**Table 3:** Characteristics of metabolic parameters factors before and after intervention^a^.

	Intervention group (*n*=86)				Mean percent reduction (%)	*p* for trend^b^	*p* ^c^	Control group (*n*=88)				Mean percent reduction (%)	*p* for trend^b^	*p* ^c^	*p* ^d^
	Time 1	Time 2	Time 3	Time 4				Time 1	Time 2	Time 3	Time 4				
SBP (mmHg)															
Male	136.6 ± 16.4	130.8 ± 15.9	133.3 ± 15.5	130.3 ± 15.0	−3.7 ± 6.9	<0.001	<0.001	132.6 ± 12.3	131.7 ± 9.6	130.0 ± 12.3	129.7 ± 12.7	−1.3 ± 7.4	0.184	0.104	0.923
Female	121.7 ± 15.2	122.3 ± 14.0	123.8 ± 19.7	121.5 ± 14.7	1.3 ± 18.4	0.678	0.954	120.1 ± 17.1	122.3 ± 15.7	123.7 ± 15.6	123.6 ± 16.4	3.2 ± 11.1	0.084	0.039	0.407
Total	128.9 ± 17.4	126.5 ± 15.4	128.4 ± 18.3	125.8 ± 15.4	−1.1 ± 14.4	0.078	0.055	125.2 ± 16.4	126.2 ± 14.2	126.3 ± 14.6	126.1 ± 15.2	1.4 ± 9.9	0.760	0.479	0.695

DBP (mmHg)															
Male	89.6 ± 11.6	85.3 ± 9.6	86.2 ± 10.4	83.8 ± 9.4	−5.3 ± 7.7	<0.001	<0.001	88.6 ± 11.9	86.1 ± 8.3	85.7 ± 8.8	83.8 ± 9.7	−3.9 ± 10.4	0.024	0.012	0.755
Female	79.5 ± 11.0	79.4 ± 10.7	79.8 ± 11.4	76.3 ± 10.4	−2.5 ± 16.7	0.060	0.053	77.9 ± 12.9	78.4 ± 11.8	79.5 ± 10.7	79.2 ± 11.9	2.0 ± 12.8	0.527	0.285	0.228
Total	84.4 ± 12.3	82.3 ± 10.5	82.9 ± 11.3	79.9 ± 10.6	−3.8 ± 13.3	<0.001	<0.001	82.3 ± 13.5	81.8 ± 11.1	82.0 ± 10.4	81.1 ± 11.2	−0.4 ± 12.1	0.599	0.271	0.368

GLU (mmol/L)															
Male	5.8 ± 1.2	5.6 ± 1.1	5.4 ± 1.3	5.5 ± 1.5	−5.1 ± 10.4	0.011	0.006	5.4 ± 1.1	5.6 ± 0.9	5.5 ± 0.9	5.4 ± 1.2	−0.7 ± 10.6	0.079	0.670	0.743
Female	5.5 ± 1.7	5.3 ± 1.2	5.3 ± 1.5	5.2 ± 1.2	−4.4 ± 9.4	0.015	0.011	5.5 ± 1.2	5.5 ± 1.0	5.4 ± 1.0	5.3 ± 1.3	−4.2 ± 9.0	0.090	0.004	0.733
Total	5.6 ± 1.5	5.4 ± 1.2	5.4 ± 1.4	5.3 ± 1.3	−4.7 ± 9.8	<0.001	<0.001	5.5 ± 1.1	5.5 ± 1.0	5.4 ± 1.0	5.4 ± 1.3	−2.7 ± 9.8	0.033	0.020	0.964

TC (mmol/L)															
Male	4.9 ± 1.0	4.8 ± 1.0	4.7 ± 1.0	4.9 ± 1.0	0.5 ± 17.6	0.190	0.448	4.7 ± 0.9	4.8 ± 0.7	4.7 ± 0.8	4.8 ± 0.8	2.4 ± 9.9	0.462	0.302	0.667
Female	4.8 ± 0.8	4.7 ± 0.8	4.6 ± 1.0	4.7 ± 0.8	−0.7 ± 8.6	0.042	0.146	4.6 ± 0.9	4.6 ± 0.9	4.6 ± 1.0	4.7 ± 1.0	3.9 ± 12.2	0.240	0.558	0.984
Total	4.9 ± 0.9	4.8 ± 0.9	4.7 ± 1.0	4.8 ± 0.9	−0.2 ± 13.3	0.007	0.177	4.6 ± 0.9	4.7 ± 0.9	4.7 ± 0.9	4.7 ± 0.9	3.3 ± 11.3	0.200	0.042	0.734

TG (mmol/L)															
Male	1.9 ± 0.8	1.9 ± 1.3	2.0 ± 1.1	1.9 ± 1.2	−3.8 ± 35.8	0.585	0.653	2.4 ± 1.4	1.9 ± 1.4	2.1 ± 1.3	2.0 ± 1.1	−11.1 ± 34.6	0.015	0.003	0.848
Female	1.5 ± 0.7	1.3 ± 0.5	1.3 ± 0.6	1.4 ± 0.9	−1.6 ± 38.3	0.425	0.396	1.5 ± 0.8	1.4 ± 0.8	1.4 ± 09	1.4 ± 1.0	3.3 ± 39.1	0.710	0.768	0.705
Total	1.7 ± 0.8	1.6 ± 1.0	1.7 ± 1.0	1.6 ± 1.1	−2.6 ± 36.9	0.566	0.397	1.9 ± 1.2	1.6 ± 1.1	1.7 ± 1.1	1.7 ± 1.1	−3.0 ± 37.7	0.028	0.014	0.903

HDL-C (mmol/L)															
Male	1.2 ± 0.4	1.4 ± 0.6	1.1 ± 0.3	1.2 ± 0.2	9.2 ± 38.4	0.023	0.543	1.1 ± 0.3	1.5 ± 0.8	1.2 ± 0.4	1.1 ± 0.2	5.7 ± 11.4	0.006	0.403	0.785
Female	1.3 ± 0.2	1.4 ± 0.4	1.2 ± 0.2	1.3 ± 0.3	−2.0 ± 11.5	0.111	0.451	1.2 ± 0.2	1.3 ± 0.5	1.3 ± 0.3	1.3 ± 0.2	6.4 ± 14.4	0.188	0.019	0.438
Total	1.2 ± 0.3	1.4 ± 0.5	1.2 ± 0.3	1.2 ± 0.3	3.0 ± 27.4	0.004	0.747	1.2 ± 0.3	1.4 ± 0.6	1.2 ± 0.4	1.2 ± 0.3	6.1 ± 13.1	0.003	0.021	0.584

LDL-C (mmol/L)															
Male	3.0 ± 0.8	3.1 ± 0.8	2.9 ± 0.6	3.0 ± 0.7	1.7 ± 18.7	0.236	0.857	2.8 ± 0.6	3.0 ± 0.6	2.8 ± 0.6	2.9 ± 0.6	4.5 ± 18.5	0.003	0.004	0.648
Female	2.9 ± 0.6	3.1 ± 0.6	2.8 ± 0.7	2.9 ± 0.6	−0.3 ± 10.7	<0.001	0.132	2.7 ± 0.7	2.9 ± 0.7	2.8 ± 0.7	2.9 ± 0.8	6.7 ± 19.3	0.007	0.004	0.681
Total	3.0 ± 0.7	3.1 ± 0.7	2.9 ± 0.7	2.9 ± 0.6	0.6 ± 14.7	0.001	0.385	2.8 ± 0.7	3.0 ± 0.7	2.8 ± 0.6	2.9 ± 0.7	5.7 ± 18.9	<0.001	<0.001	0.992

^a^Data are given as means (SD); ^b^data were analyzed by repeated measurements; ^c^data analyzed by paired-samples *T*-test between time 1 and time 4; ^d^data analyzed by *T*-test between the intervention group and control group at time 4. SPB: systolic blood pressure; DBP: diastolic blood pressure; GLU: fasting glucose; TC: total cholesterol; TG: triglycerides; HDL-C: high-density lipoprotein cholesterol; LDL-C: low-density lipoprotein cholesterol. *p* < 0.05 indicates statistical significance.

## Data Availability

The database of participants' data used to support the findings of this study are included within the Supplementary File S2. Database of participants. Individual identified participant data are available, including basic information, body composition, and metabolic parameters. Other documents, such as study protocol and statistical analysis plan, are not available. All the readers can review the database with attachment File S2.

## Supplementary Materials

Supplementary Materials Table S1: ingredients of protein powder and fibergy; Table S2: dietary characteristics before and after the meal replacement intervention; Table S3: additional body composition characteristic before and after intervention; File S1: consort-2010-checklist. File S2: database of participants.
