# Supplementary material for: Effects of a Meal Replacement on Body Composition and Metabolic Parameters among Subjects with Overweight or Obesity
Source: J Obes. 2018 Dec 26;2018:2837367. doi: 10.1155/2018/2837367 (PMC6327254; doi:10.1155/2018/2837367)
Supplement: Supplementary Materials — Table S1: ingredients of protein powder and fibergy; Table S2: dietary characteristics before and after the meal replacement intervention; Table S3: additional body composition characteristic before and after intervention; File S1: consort-2010-checklist. File S2: database of participants. [file 2837367.f1.zip › 2837367.f1/Supplementary tables_JOBE_2598330.docx]

Table S1. Ingredients of protein powder

| Item | Content(g/100g) | NRV% |
| --- | --- | --- |
| Energy | 402 kcal | 20 |
| Protein | 25.0 g | 42 |
| Fat | 12.3 g | 21 |
| Carbohydrate | 42.2 g | 14 |
| Dietary fiber | 10.5 g | 42 |
| Na | 590mg | 30 |
| Vitamin A | 470μg RE | 59 |
| Vitamin E | 14mg α-TE | 100 |
| Vitamin B1 | 1mg | 71 |
| Vitamin B2 | 1.5 mg | 107 |
| Vitamin B6 | 1.5 mg | 107 |
| Vitamin C | 100.0 mg | 100 |
| Mg | 155 mg | 52 |
| Ca | 350 mg | 44 |
| Zn | 67 mg | 67 |

Table S1. Ingredients of Fibergy

| Item | Content(g/100g) | NRV% |
| --- | --- | --- |
| Energy | 190 kcal | 9 |
| Protein | 0.7g | 1 |
| Fat | 0g | 0 |
| Carbohydrate | 7.5 g | 3 |
| Dietary fiber | 82 g | 328 |
| Na | 170mg | 9 |

NRV: Nutrient Reference Values;

Participants consumed 90g protein powder and 14g fibergy each day during the intervention period.

TableS2. Dietary characteristics before and after the meal replacement intervention ^a^

|  |  | Baseline | | | Post | | |  |  |
| --- | --- | --- | --- | --- | --- | --- | --- | --- | --- |
|  |  | intervention group  (n=86) | control group  (n=88) | *p*^b^ | intervention group  (n=86) | control group  (n=88) | *p*^b^ | *p*^c^ | *p*^d^ |
| Total energy intake (g/d) | Male | 2162±390 | 2108±306 | 0.495 | 1822±185 | 2073±235 | <0.001 | <0.001 | 0.065 |
|  | Female | 1832±294 | 1911±399 | 0.284 | 1684±156 | 1891±192 | <0.001 | <0.001 | 0.389 |
|  | Total | 1995±380 | 1995±373 | 0.997 | 1751±184 | 1971±229 | <0.001 | <0.001 | 0.660 |
| Total protein intake (g/d) | Male | 78.0±20.4 | 76.8±17.5 | 0.772 | 85.5±10.5 | 78.1±13.1 | 0.008 | 0.002 | 0.807 |
|  | Female | 65.2±17.0 | 67.3±18.9 | 0.580 | 81.2±11.5 | 74.9±13.5 | 0.020 | 0.004 | 0.004 |
|  | Total | 71.6±17.7 | 71.4±18.8 | 0.943 | 83.2±11.2 | 76.3±13.4 | <0.001 | <0.001 | 0.042 |
| Total fat intake (g/d) | Male | 89.0±17.2 | 85.3±16.4 | 0.322 | 70.6±8.9 | 68.5±11.1 | 0.362 | <0.001 | <0.001 |
|  | Female | 79.9±17.8 | 80.0±20.2 | 0.971 | 66.5±8.0 | 63.8±11.2 | 0.198 | <0.001 | <0.001 |
|  | Total | 84.4±18.0 | 82.3±18.7 | 0.445 | 68.5±8.7 | 65.9±11.3 | 0.096 | <0.001 | <0.001 |
| Total carbohydrates intake (g/d) | Male | 260.0±43.4 | 256.0±44.4 | 0.719 | 209.1±27.2 | 281.9±29.5 | <0.001 | <0.001 | 0.006 |
|  | Female | 214.5±41.5 | 231.2±58.4 | 0.118 | 188.7±26.7 | 255.7±28.6 | <0.001 | <0.001 | <0.001 |
|  | Total | 236.8±47.9 | 241.8±54.0 | 0.517 | 198.5±28.7 | 267.2±31.6 | <0.001 | <0.001 | <0.001 |
| Total fiber intake (g/d) | Male | 10.0±3.7 | 9.6±3.1 | 0.560 | 31.8±2.6 | 14.5±2.3 | <0.001 | <0.001 | <0.001 |
|  | Female | 9.3±3.1 | 10.3±4.3 | 0.225 | 31.9±2.7 | 14.8±2.4 | <0.001 | <0.001 | <0.001 |
|  | Total | 9.7±3.4 | 10.0±3.8 | 0.576 | 31.9±2.7 | 14.6±2.3 | <0.001 | <0.001 | <0.001 |
| Energy from protein (%) | Male | 14.3±2.3 | 14.5±2.1 | 0.777 | 18.8±1.5 | 15.0±1.6 | <0.001 | <0.001 | 0.259 |
|  | Female | 14.1±2.5 | 14.0±2.2 | 0.808 | 19.3±2.0 | 15.8±1.9 | <0.001 | <0.001 | <0.001 |
|  | Total | 14.2±2.4 | 14.2±2.2 | 0.948 | 19.0±1.8 | 15.4±1.8 | <0.001 | <0.001 | <0.001 |
| Energy from fat (%) | Male | 37.1±3.9 | 36.5±5.4 | 0.550 | 34.9±3.3 | 29.7±3.4 | <0.001 | 0.070 | <0.001 |
|  | Female | 39.1±5.8 | 27.7±6.0 | 0.250 | 35.6±3.5 | 30.3±4.3 | <0.001 | <0.001 | <0.001 |
|  | Total | 38.1±5.0 | 37.2±5.8 | 0.252 | 35.3±3.4 | 30.6±4.5 | <0.001 | <0.001 | <0.001 |
| Energy from carbohydrates (%) | Male | 48.4±4.6 | 48.7±5.6 | 0.771 | 45.8±3.0 | 54.6±4.3 | <0.001 | 0.005 | <0.001 |
|  | Female | 47.1±7.2 | 48.5±6.8 | 0.324 | 44.9±4.5 | 54.2±5.1 | <0.001 | 0.074 | <0.001 |
|  | Total | 47.7±6.1 | 48.6±6.2 | 0.350 | 45.3±3.9 | 54.4±4.7 | <0.001 | 0.001 | <0.001 |
| Milk Consumption(g/d) | Male | 124.3±81.4 | 122.5±99.0 | 0.927 | 134.6±49.8 | 166.7±57.4 | 0.01 | 0.034 | 0.007 |
|  | Female | 140.3±89.5 | 132.7±79.4 | 0.663 | 132.2±62.8 | 167.6±69.2 | 0.012 | 0.016 | 0.205 |
|  | Total | 132.4±85.5 | 128.3±88.2 | 0.757 | 133.3±56.5 | 137.2±63.0 | <0.001 | 0.001 | 0.005 |
| Meat consumption(g/d) | Male | 183.5±75.8 | 178.2±64.9 | 0.739 | 107.2±22.9 | 166.9±20.7 | <0.001 | <0.001 | 0.105 |
|  | Female | 139.8±72.6 | 131.3±74.4 | 0.578 | 89.8±21.3 | 132.0±23.8 | <0.001 | <0.001 | 0.743 |
|  | Total | 161.4±77.0 | 151.3±75.5 | 0.378 | 98.2±23.6 | 147.3±28.3 | <0.001 | <0.001 | 0.425 |
| Fish consumption(g/d) | Male | 29.1±29.0 | 23.1±29.2 | 0.360 | 27.2±15.5 | 31.9±18.9 | 0.232 | 0.050 | 0.333 |
|  | Female | 26.1±28.6 | 25.7±31.4 | 0.944 | 30.0±16.1 | 45.4±25.8 | <0.001 | 0.896 | 0.001 |
|  | Total | 27.6±28.7 | 24.6±30.3 | 0.501 | 28.6±15.8 | 39.5±23.9 | 0.001 | <0.001 | 0.001 |
| Legume consumption(g/d) | Male | 8.4±11.9 | 4.5±7.7 | 0.089 | 24.7±8.6 | 34.4±8.4 | <0.001 | <0.001 | <0.001 |
|  | Female | 5.2±8.7 | 6.7±9.9 | 0.439 | 21.9±9.5 | 35.5±7.3 | <0.001 | <0.001 | <0.001 |
|  | Total | 6.8±10.5 | 5.8±9.0 | 0.491 | 23.3±9.1 | 35.0±7.8 | <0.001 | <0.001 | <0.001 |
| Cereal consumption(g/d) | Male | 291.8±53.1 | 290.8±60.9 | 0.943 | 127.3±21.3 | 273.0±42.2 | <0.001 | <0.001 | <0.001 |
|  | Female | 213.5±46.4 | 234.7±60.4 | 0.060 | 99.3±20.8 | 244.8±36.5 | <0.001 | <0.001 | 0.068 |
|  | Total | 252.2±63.3 | 258.7±66.4 | 0.505 | 112.8±25.2 | 257.1±41.3 | <0.001 | <0.001 | <0.001 |
| Vegetable consumption(g/d) | Male | 255.6±168.8 | 222.9±128.5 | 0.336 | 253.2±87.9 | 286.8±75.9 | 0.075 | 0.110 | 0.026 |
|  | Female | 263.7±133.0 | 240.2±147.2 | 0.415 | 279.6±75.1 | 280.5±75.6 | 0.951 | 0.436 | 0.058 |
|  | Total | 259.8±150.9 | 232.8±139.0 | 0.219 | 266.8±82.1 | 283.3±75.3 | 0.175 | 0.718 | 0.003 |
| Fruits consumption(g/d) | Male | 319.8±229.0 | 317.1±264.3 | 0.961 | 361.0±138.5 | 388.3±134.2 | 0.379 | 0.526 | 0.007 |
|  | Female | 290.4±172.6 | 395.7±330.0 | 0.060 | 370.9±148.6 | 410.3±103.0 | 0.141 | 0.560 | 0.013 |
|  | Total | 304.9±201.8 | 362.2±304.6 | 0.145 | 366.1±143.0 | 400.5±117.4 | 0.087 | 0.912 | <0.001 |
| Nuts consumption(g/d) | Male | 4.0±4.8 | 3.8±5.1 | 0.881 | 4.4±4.4 | 6.4±3.4 | 0.035 | 0.436 | 0.002 |
|  | Female | 4.8±4.7 | 6.0±7.0 | 0.329 | 6.3±7.2 | 6.8±3.1 | 0.552 | 0.922 | 0.022 |
|  | Total | 4.4±4.7 | 5.0±6.3 | 0.419 | 5.4±6.0 | 6.6±3.8 | 0.089 | 0.639 | 0.002 |
| Beverage consumption(mL/d) | Male | 84.7±89.1 | 103.0±97.7 | 0.380 | 60.5±56.2 | 107.8±69.1 | 0.001 | <0.001 | 0.942 |
|  | Female | 134.0±133.0 | 92.4±111.5 | 0.101 | 64.5±55.3 | 101.0±70.4 | 0.07 | 0.006 | 0.821 |
|  | Total | 109.7±115.5 | 97.0±105.3 | 0.447 | 62.6±55.4 | 104.0±66.2 | <0.001 | <0.001 | 0.884 |
| Alcohol intake(g/d) | Male | 9.3±24.4 | 8.4±23.6 | 0.877 | 4.7±9.7 | 9.6±14.9 | 0.088 | 0.339 | 0.904 |
|  | Female | 2.9±7.5 | 4.0±10.9 | 0.561 | 3.3±7.8 | 2.5±5.5 | 0.598 | 0.560 | 0.425 |
|  | Total | 6.0±18.0 | 5.9±17.5 | 0.960 | 3.9±8.7 | 5.6±11.2 | 0.284 | 0.269 | 0.720 |
| Physical activity (MET-min/d) | Male | 463.1±21.8 | 459.1±144.7 | 0.885 | 463.7±158.6 | 458.8±171.2 | 0.896 | 0.983 | 0.994 |
|  | Female | 440.9±134.0 | 459.4±169.7 | 0.569 | 436.3±160.5 | 438.8±182.1 | 0.933 | 0.894 | 0.527 |
|  | Total | 451.6±134.6 | 459.3±158.5 | 0.749 | 449.6±159.2 | 447.4±176.8 | 0.950 | 0.926 | 0.639 |

a: Data are given as means (SD);

b: Data were analyzed by T-test;

c: Data analyzed by Paired-Samples T-test between intervention group at baseline and post intervention;

d: Data analyzed by Paired-Samples T-test between the control group at baseline and post intervention;

p < 0.05 indicates statistical significance.

Table S3. Additional body composition characteristic before and after intervention ^a^

|  |  | Intervention group (n=86) | | | | |  |  | Control group(n=88) | | | | |  |  |  |
| --- | --- | --- | --- | --- | --- | --- | --- | --- | --- | --- | --- | --- | --- | --- | --- | --- |
|  |  | Time1 | Time2 | Time 3 | Time4 | Mean percent reduction(%) | *p* for trends ^b^ | *p*^c^ | Time1 | Time2 | Time 3 | Time4 | Mean percent reduction(%) | *p* for trends ^b^ | *p*^c^ | *p*^d^ |
| ICW  (kg) | Male | 28.4±3.1 | 28.0±2.9 | 27.7±3.0 | 27.9±3.0 | -1.7±2.4 | <0.001 | <0.001 | 27.7±2.9 | 27.8±3.1 | 27.5±3.1 | 27..7±3.3 | 0.7±4.2 | 0.139 | 0.174 | 0.874 |
|  | Female | 20.0±1.9 | 19.6±2.0 | 19.6±1.8 | 19.7±1.8 | -1.2±3.5 | <0.001 | 0.016 | 20.0±2.0 | 20.0±2.1 | 19.9±2.1 | 20.0±2.1 | -0.3±2.8 | 0.260 | 0.477 | 0.476 |
|  | Total | 23.9±4.9 | 23.5±4.8 | 23.3±4.8 | 23.5±4.8 | -1.4±3.0 | <0.001 | <0.001 | 23.3±4.5 | 23.3±4.7 | 23.1±4.6 | 23.3±4.8 | 0.1±3.5 | 0.083 | 0.512 | 0.985 |
| ECW  (kg) | Male | 17.4±1.9 | 16.9±1.8 | 16.8±1.8 | 16.8±1.7 | -3.0±2.9 | <0.001 | 0.090 | 16.7±1.8 | 16.6±1.9 | 16.5±1.9 | 16.6±2.0 | -0.8±3.5 | 0.060 | 0.096 | 0.783 |
|  | Female | 12.5±1.2 | 12.1±1.2 | 12.1±1.2 | 12.1±1.2 | -2.7±3.3 | <0.001 | <0.001 | 12.4±1.2 | 12.3±1.3 | 12.2±1.3 | 12.2±1.2 | -1.8±3.2 | <0.001 | <0.001 | 0.816 |
|  | Total | 14.7±2.9 | 14.3±2.8 | 14.3±2.8 | 14.3±2.8 | -2.8±3.1 | <0.001 | <0.001 | 14.2±2.6 | 14.1±2.7 | 14.0±2.6 | 14.1±2.7 | -1.4±3.4 | <0.001 | <0.001 | 0.752 |
| TW  (kg) | Male | 45.8±5.0 | 44.9±4.7 | 44.6±4.8 | 44.7±4.7 | -2.0±2.4 | <0.001 | <0.001 | 44.4±4.6 | 44.5±5.0 | 44.0±5.1 | 44.5±5.3 | 0.2±3.8 | 0.166 | 0.675 | 0.998 |
|  | Female | 32.4±3.1 | 31.7±3.2 | 31.7±3.0 | 31.8±3.2 | -1.8±3.3 | <0.001 | <0.001 | 32.4±3.1 | 32.2±3.3 | 32.1±3.3 | 32.1±3.3 | -0.9±2.9 | 0.020 | 0.041 | 0.592 |
|  | Total | 38.6±7.8 | 37.8±7.7 | 37.6±7.6 | 37.8±7.6 | -2.0±2.9 | <0.001 | <0.001 | 37.5±7.1 | 37.4±7.3 | 37.1±7.2 | 37.4±7.5 | -2.0±2.9 | 0.026 | 0.421 | 0.918 |
| Protein  (kg) | Male | 12.3±1.3 | 12.1±1.3 | 12.0±1.3 | 12.1±1.3 | -1.6±2.1 | <0.001 | <0.001 | 12.0±1.2 | 12.0±1.3 | 11.9±1.4 | 12.1±1.4 | 0.8±4.3 | 0.124 | 0.244 | 0.924 |
|  | Female | 8.6±0.8 | 8.5±0.8 | 8.5±0.8 | 8.5±0.9 | -1.1±3.5 | <0.001 | 0.021 | 8.6±0.8 | 8.6±0.9 | 8.6±0.9 | 8.6±0.9 | -0.0±2.9 | 0.386 | 1 | 0.477 |
|  | Total | 10.3±2.1 | 10.1±2.1 | 10.1±2.1 | 10.2±2.1 | -1.3±2.9 | 0.001 | <0.001 | 10.0±1.9 | 10.1±2.0 | 10.0±2.0 | 10.1±2.1 | 0.3±3.5 | 0.057 | 0.310 | 0.994 |
| Minerals  (kg) | Male | 4.2±0.5 | 4.1±0.5 | 4.1±0.5 | 4.1±0.5 | -0.9±2.3 | <0.001 | 0.019 | 4.1±0.5 | 4.1±0.5 | 4.1±0.5 | 4.1±0.6 | 0.5±5.9 | 0.348 | 0.591 | 0.989 |
|  | Female | 3.1±0.3 | 3.0±0.3 | 3.0±0.3 | 3.0±0.3 | -1.4±4.2 | <0.001 | 0.023 | 3.1±0.3 | 3.1±0.3 | 3.1±0.3 | 3.1±0.3 | -1.2±3.3 | 0.004 | 0.016 | 0.583 |
|  | Total | 3.6±0.7 | 3.5±0.7 | 3.5±0.7 | 3.5±0.7 | -1.2±3.4 | <0.001 | <0.001 | 3.5±0.6 | 3.5±0.7 | 3.5±0.6 | 3.5±0.7 | -0.5±4.7 | 0.151 | 0.546 | 0.983 |
| BMR  (kcal/day) | Male | 1714±148 | 1690±139 | 1681±143 | 1690±139 | -1.3±1.7 | <0.001 | <0.001 | 1676±140 | 1679±147 | 1666±149 | 1681±156 | 0.2±3.1 | 0.157 | 0.571 | 0.922 |
|  | Female | 1324±93 | 1304±94 | 1302±88 | 1307±94 | -1.2±2.4 | <0.001 | 0.001 | 1324±92 | 1319±99 | 1315±99 | 1317±98 | -0.5±2.0 | 0.033 | 0.085 | 0.557 |
|  | Total | 1503±230 | 1482±226 | 1477±223 | 1484±225 | -1.3±2.1 | <0.001 | <0.001 | 1473±208 | 1470±216 | 1463±213 | 1471±220 | -0.2±2.6 | 0.038 | 0.660 | 0.897 |

a: Data are given as means (SD);

b: Data were analyzed by repeated measurements;

c: Data analyzed by Paired-Samples T-test between time 1 and time 4;

d: Data analyzed by T-test between intervention group and control group at time 4.

ICW: intracellular water; ECW: extracellular water; TW: total water; BMR: basal metabolic rate.

p < 0.05 indicates statistical significance.
